# Efficient Music Genre Recognition Using ECAS-CNN: A Novel Channel-Aware Neural Network Architecture

**DOI:** 10.3390/s24217021

**Published:** 2024-10-31

**Authors:** Yang Ding, Hongzheng Zhang, Wanmacairang Huang, Xiaoxiong Zhou, Zhihan Shi

**Affiliations:** 1 College of Education, Bashkir State Pedagogical University, Ufa 450000, Russia; din003376@gmail.com (Y.D.); huangwanmacairang@gmail.com (W.H.); 2 College of Electrical Engineering and Control Science, Nanjing Tech University, Nanjing 211899, China; zhouxx@njtech.edu.cn (X.Z.); szh@njtech.edu.cn (Z.S.)

**Keywords:** music genre classification, channel-aware convolutional neural network, ECA module, deep learning, GTZAN dataset, music information retrieval

## Abstract

In the era of digital music proliferation, music genre classification has become a crucial task in music information retrieval. This paper proposes a novel channel-aware convolutional neural network (ECAS-CNN) designed to enhance the efficiency and accuracy of music genre recognition. By integrating an adaptive channel attention mechanism (ECA module) within the convolutional layers, the network significantly improves the extraction of key musical features. Extensive experiments were conducted on the GTZAN dataset, comparing the proposed ECAS-CNN with traditional convolutional neural networks. The results demonstrate that ECAS-CNN outperforms conventional methods across various performance metrics, including accuracy, precision, recall, and F1-score, particularly in handling complex musical features. This study validates the potential of ECAS-CNN in the domain of music genre classification and offers new insights for future research and applications.

## 1. Introduction

In the current era of digital music proliferation, Music Information Retrieval (MIR) has emerged as a pivotal domain within music technology research. Music genre classification, as a core task of MIR, is integral not only to the development of music recommendation systems and the creation of playlists but also to the intelligent analysis and management of music files. With the rapid expansion of online music services, such as Spotify and Apple Music, the accuracy of genre classification directly influences both the user experience and the effectiveness of copyright management [1,2].

The classification of music genres is based on the analysis of various features within the musical signal, including but not limited to melody, rhythm, harmony, tempo, and timbre. The extraction and analysis of these musical features are crucial for understanding the intrinsic characteristics of different music genres. However, the complexity of musical signals and the subtle differences between music styles pose significant challenges to automatic music classification. For instance, tracks within the same genre may exhibit considerable variation due to differences in the artist’s style, recording techniques, or the historical period in which they were produced [3].

Additionally, with the proliferation of mobile internet and smart devices, users’ expectations for music recommendation systems have steadily increased, driving researchers to develop more efficient and intelligent music classification algorithms. In recent years, the introduction of deep learning techniques has provided novel solutions for addressing these complex data challenges. By utilizing Convolutional Neural Networks (CNNs), Recurrent Neural Networks (RNNs), and, more recently, Transformer models, researchers have been able to learn deep patterns from vast amounts of music data, thereby significantly improving the accuracy of music classification [4,5]. Nonetheless, music genre classification still faces unique challenges. For instance, issues such as handling noise and distortion in musical signals and overcoming the crossover influences between different music styles remain unresolved. Furthermore, the classification of musical emotions and emotion-based music recommendation have also emerged as research hotspots [6], necessitating algorithms that can not only identify the genre of music but also comprehend the emotional content it conveys.

### 1.1. Research Questions and Hypotheses

This study aims to address key issues in contemporary music genre classification by proposing a novel Efficient Channel-Aware Convolutional Neural Network (ECAS-CNN) architecture. Specifically, the research questions of this study can be summarized as follows:

**Hypothesis 1:**  *By introducing an adaptive channel attention mechanism (ECA module), the network’s capability to capture critical features in music signals can be enhanced, thereby improving classification accuracy.*

**Hypothesis 2:**  *Compared to traditional convolutional neural networks, ECAS-CNN exhibits advantages in reducing computational complexity and improving model efficiency, particularly when processing large-scale music datasets.*

### 1.2. Background in the Field of Music Information Retrieval (MIR)

Music information retrieval (MIR) is an interdisciplinary field concerned with the processing, analysis, and retrieval of music data, encompassing areas such as audio signal processing, machine learning, and deep learning. In recent years, with the rapid growth of music streaming platforms and digital music, genre classification within the MIR field has become a prominent research topic. Researchers employ various audio features, such as Mel spectrograms and MFCC (Mel Frequency Cepstral Coefficients), to convert one-dimensional audio signals into two-dimensional feature maps suitable for deep learning models, greatly enhancing the accuracy and efficiency of MIR systems.

## 2. Related Work

Ziyan Zhao et al. conducted an in-depth analysis of the GTZAN dataset using Support Vector Machines (SVM). They discovered that segmenting the audio into 3-s intervals significantly enhanced the classification performance of SVM. This segmentation strategy allows the model to more effectively capture key features within the musical signal, particularly when employing a linear kernel. SVM outperformed other traditional algorithms such as logistic regression and random forests, highlighting the importance of audio preprocessing in improving classification accuracy. Different temporal segmentation strategies can substantially influence the performance of classification models [7]. Mitt Shah et al. explored the application of deep learning techniques in music genre classification. Their study utilized the GTZAN dataset to train various deep learning models and ultimately found that CNNs excelled in handling this task. Compared to traditional machine learning methods, CNNs are capable of extracting deeper features from the data, giving them a distinct advantage in music genre classification. The findings underscore the powerful capability of deep learning, particularly CNNs, in processing complex and high-dimensional data [8]. In another study, M.V.S.L. Jahnavi et al. conducted a comprehensive comparison of several machine learning models and contrasted their performance with that of CNNs. The results revealed that CNNs achieved an accuracy of 94.87% on the GTZAN dataset, significantly outperforming traditional machine learning methods. This further attests to the superiority of deep learning approaches, particularly CNNs, in music classification tasks, and illustrates the limitations of traditional algorithms when faced with complex musical data [9]. Xiaoxiao Deng (2024) proposed an improved deep convolutional neural network, DenseNet-II, which achieved a classification accuracy of 98.2% on the GTZAN dataset, demonstrating the immense potential of deep learning techniques in this field. DenseNet-II enhances the transmission and reuse of features through dense connectivity layers, allowing the network to learn the complex characteristics of musical signals more efficiently without increasing model complexity [10]. Rudresh Pillai et al. designed a sequential model for music genre classification by combining Mel spectrograms with the GTZAN dataset, ultimately achieving an accuracy of 94%. This study further validates the effectiveness of spectrograms in music signal processing, especially when integrated with deep learning models. As a two-dimensional representation of musical signals, Mel spectrograms capture frequency characteristics, enabling the model to more accurately distinguish between different music genres [11]. S. J et al. combined the k-nearest neighbors (KNN) algorithm with Principal Component Analysis (PCA) and achieved a maximum classification accuracy of 77.41% on the GTZAN dataset. PCA effectively reduced the dimensionality of the data, thereby enhancing the performance of the classifier. This approach exemplifies the strategy of improving model efficiency and accuracy by reducing data dimensionality in high-dimensional data processing [12]. Zhiwei Liu et al. developed a Locally Activated Gated Neural Network (LGNet) and tested it on the GTZAN dataset. The results indicated that this network outperformed existing music genre classification methods, demonstrating its significant potential in automated music classification. LGNet’s local activation mechanism effectively increases the model’s sensitivity to different music genres, resulting in more precise classification [13]. S. Patil et al. proposed a novel mathematical model utilizing deep neural networks for the classification of music and rhythm genres and validated its effectiveness across multiple datasets, including the GTZAN dataset. Their findings highlight the promising prospects of deep learning technologies in music genre classification, particularly in handling complex rhythm patterns [14]. N. Srivastava et al. employed a Convolutional Recurrent Neural Network (CNN-LSTM) for music genre classification on the GTZAN dataset, achieving an accuracy of 87.5%. This study further validates the advantages of deep learning, particularly the combination of CNN and LSTM, in tackling complex datasets [15]. Mousumi Chaudhury et al. conducted large-scale music genre analysis and classification using the Apache Spark platform and validated the effectiveness of the Random Forest algorithm on the GTZAN dataset, establishing it as the best classifier. This study showcases the potential of big data technologies in music classification tasks, particularly the efficiency of the Apache Spark platform and the robustness of the Random Forest algorithm in handling large datasets [16].

Through these various studies, it is evident that while traditional machine learning and deep learning techniques have made significant strides in music genre classification, challenges such as handling noise, distortion, and subtle inter-genre differences persist. Additionally, the need for more efficient and accurate models that can adapt to the growing complexity of musical data is increasingly apparent. Building on this foundation, our research introduces the Efficient Channel Attention Convolutional Neural Network (ECAS-CNN), which integrates an adaptive channel attention mechanism to enhance feature extraction and classification accuracy. This novel approach not only addresses the existing challenges in music genre classification but also sets a new benchmark for performance, as demonstrated by its superior results on the GTZAN dataset.

This paper is structured into four main chapters, providing a detailed discussion of the research on music genre classification, as well as the methods and experimental results of the proposed approach. Section 1 reviews the cutting-edge research related to this study, with a particular focus on current mainstream music genre classification methods, evaluating their effectiveness and applications. Section 3 elaborates on the network architecture and methodology developed in this study, specifically detailing how the Efficient Channel Attention (ECA) module is integrated into the Convolutional Neural Network to enhance feature extraction and classification accuracy. Section 4 presents a series of experiments conducted using the GTZAN music dataset, along with an analysis of the results to validate the effectiveness of the proposed method. Finally, Section 5 summarizes the key research findings of this paper and offers insights into future research directions.

## 3. Methodology

### 3.1. Mel-Spectrogram Model

Musical data, in essence, are a series of time-varying sound signals, typically represented in the form of waveforms. These waveforms not only depict the changes in frequency and amplitude of sound over time but also implicitly contain a wealth of musical information such as pitch, rhythm, and harmony. While these one-dimensional audio signals are rich in musical features, directly applying them to complex digital signal processing and machine learning models, especially deep learning architectures like Convolutional Neural Networks (CNNs), often yields suboptimal results. This is because one-dimensional signals struggle to fully capture the spatial relationships and structural features inherent in music. Therefore, to facilitate more effective music information retrieval and classification, it is necessary to convert these one-dimensional audio signals into more informative two-dimensional data formats.

In this conversion process, Mel Frequency Cepstral Coefficients (MFCC) emerge as an invaluable tool [17]. MFCCs simulate the nonlinear perception of different frequencies by the human ear, enabling the extraction of key musical features from audio signals. Specifically, MFCC begins by decomposing the audio signal into a series of overlapping short-time frames, each representing a snapshot of sound at a given moment. Fast Fourier Transform (FFT) is then applied to each frame to analyze its frequency components, which are subsequently mapped onto the Mel scale. The Mel scale, a frequency scale based on human auditory perception, is more adept at capturing the essential characteristics of musical signals. Following this, logarithmic transformation and Discrete Cosine Transform (DCT) are performed to generate a series of coefficients that make up the final feature vector. These feature vectors not only reduce data redundancy but also enhance the classifier’s ability to distinguish between different musical styles.

**Figure 1:**
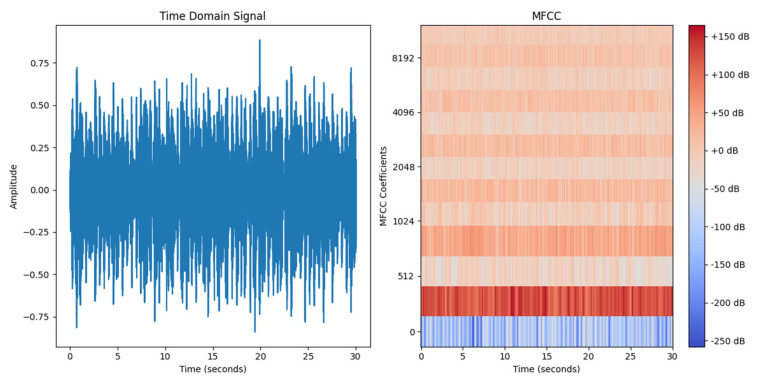
The timedomain waveform and the MFCC feature map of the audio data ((**left**): time-domain waveform, (**right**): MFCC feature map).

The audio data processed by MFCC is transformed into a richly informative two-dimensional feature map, a representation that is ideally suited for analysis by Convolutional Neural Networks (CNNs). The multi-layer convolutional structure of CNNs is capable of extracting and learning local patterns and structures from these feature maps, capturing the complex features embedded within the musical signals. This ability to extract features gives CNNs a significant advantage and efficiency in recognizing and differentiating various musical styles and content. Consequently, the combination of MFCC and CNN provides robust technical support for music classification tasks. As shown in Figure 1, the left image presents the time-domain waveform of the audio signal, while the right image displays the two-dimensional feature map generated through MFCC. This graphical representation is particularly suitable for analysis by CNNs.

### 3.2. ECA Module

The Efficient Channel Attention (ECA) module represents an enhancement and refinement of the traditional attention mechanism, specifically the Squeeze-and-Excitation (SE) module. While the SE module has been successful in improving the feature learning capabilities of neural networks, it relies heavily on fully connected (FC) layers to learn the nonlinear relationships between channels. This reliance results in higher computational complexity and an increased number of parameters, which can negatively impact the efficiency and practical effectiveness of the model, particularly when dealing with large-scale data.

To address these challenges, the ECA module introduces a more lightweight channel attention mechanism that significantly reduces computational overhead and the number of parameters while maintaining robust feature learning capabilities. Specifically, the ECA module utilizes a one-dimensional convolution operation to directly adjust channel weights, eliminating the need for fully connected layers. This approach not only effectively preserves global information but also significantly enhances the model’s ability to capture local dependencies, which is crucial for learning complex features, especially in tasks like music genre classification that involve high-dimensional feature spaces.

The music signal itself possesses a highly intricate feature structure, including multiple levels of information such as melody, rhythm, harmony, and timbre. There are intricate dependencies between these features, and they exhibit strong localized correlations in both the frequency and time domains. The ECA module, via an adaptive channel weighting mechanism, effectively enhances the model’s ability to capture salient musical features, enabling it to better extract and distinguish these complex high-dimensional features in the task of music genre classification. In music genre classification, the presence of background noise and the overlap of features between styles present a major challenge. The ECA module, through adaptive channel weight adjustment, more effectively isolates key features within the music signal, thereby mitigating the impact of noise on classification accuracy. Moreover, the ECA module demonstrates flexibility in adjusting to various music genres, enabling superior performance with diverse musical data and effectively enhancing cross-genre classification capability.

The core innovation of the ECA module lies in its adaptive selection of convolutional kernel size to better align with the dimensions of different feature maps. This adaptability is achieved through a simple yet effective function that dynamically adjusts the kernel size based on the input feature dimensions, optimizing the learning of inter-channel dependencies. This design not only enhances the module’s flexibility, allowing it to adapt to various network architectures and different task requirements, but also improves the model’s practicality and efficiency across diverse application scenarios. As a result, the ECA module effectively balances performance with computational complexity, making it particularly well-suited for deployment in resource-constrained environments.

(1) $$k=\left|\frac{log_{2}\left(c\right)}{\gamma }\frac{b}{\gamma }\right|$$

**Figure 2:**
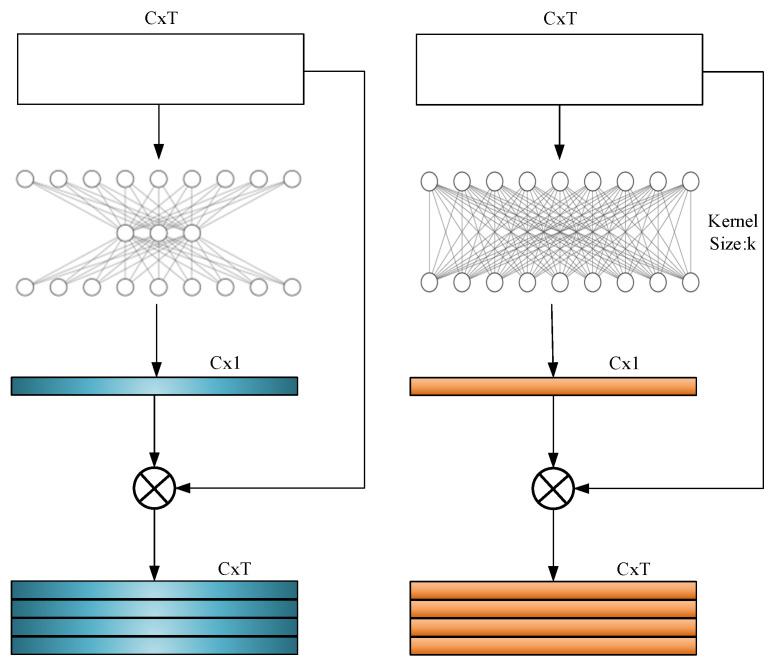
The comparison of SE and ECA network structures ((**left**): SE block, (**right**): ECA block).

Formula (1) dynamically adjusts the convolutional kernel size by taking into account the number of channels, ensuring a balance between processing efficiency and performance. Additionally, the ECA module utilizes standard layers. Conv1D operations to implement its functionality, making it easy to integrate into existing convolutional networks. As illustrated in Figure 2, by comparing the network structures of SE and ECA, it is evident how ECA enhances computational efficiency by reducing model complexity while maintaining performance.

### 3.3. Model Design

**Figure 3:**
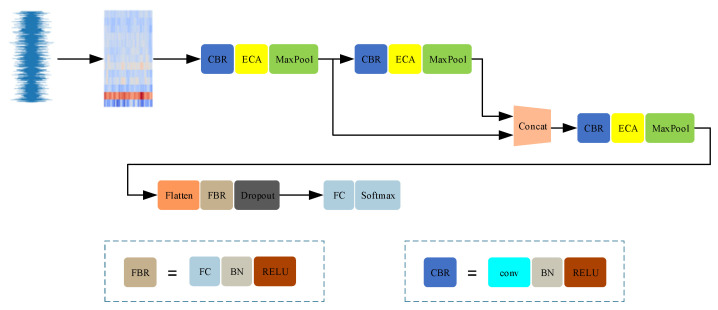
The network architecture diagram of our approach. (Different colors in the figure represent different modules).

To comprehensively capture the complex features within audio signals, the Convolutional Neural Network (CNN) proposed in this paper consists of four convolutional layers, each equipped with a custom-designed ECA module. The choice of network depth and the number of layers is based on the need to extract features at different hierarchical levels. Audio signals contain multi-level information, ranging from basic tonal qualities to complex rhythms and harmonics. An appropriately deep network facilitates the effective learning of representative features from these intricate signals, as shown in Figure 3.

The first convolutional layer primarily captures the fundamental features of the raw audio data, such as edges and simple textures, which serve as the foundation for identifying more advanced features in subsequent layers. As the network deepens, the following convolutional layers further abstract these basic features into more complex musical structures, such as melodic lines and rhythmic patterns. With each additional layer, the network’s capacity for abstraction increases, enabling the model to more accurately recognize and classify more intricate music genres.

The operations of the convolutional layers can be expressed by Formula (2).

(2) $$Y=f\left(W*X+b\right)$$

here, X represents the input feature map, W denotes the convolutional kernel weights, b refers to the bias, and f is the activation function, such as ReLU, which ensures the learning of nonlinear features.

The introduction of the ECA module after each convolutional layer is intended to enhance the model’s sensitivity to key musical features. By adaptively adjusting channel attention, the ECA module enables the network to focus more precisely on the features that are most critical for classification decisions. This fine-tuned adjustment is particularly crucial for handling the subtle nuances present in musical data. The ECA module adjusts channel weights through the following operations:

(3) $$z_{c}=\frac{1}{H\times W}\sum_{i=1}^{H}\sum_{j=1}^{W}x_{cij}$$

(4) $$Y_{c}=\sigma \left(w*z\right)\cdot x_{c}$$

here, σ represents the sigmoid function and w denotes the one-dimensional convolutional kernel. This demonstrates how global average pooling and one-dimensional convolution are utilized to adjust channel attention, thereby enhancing the model’s feature selection capability.

Additionally, an appropriate network depth helps to avoid overfitting. While deeper networks theoretically offer greater learning capacity, they also tend to increase training difficulty and require more computational resources. This model achieves a balance between performance and computational efficiency through carefully designed layers and regularization strategies such as batch normalization and dropout. The mathematical expressions for batch normalization and dropout are as follows:

(5) $${\hat{x}}_{i}=\gamma \left(\frac{x_{i}-\mu_{B}}{\sqrt{\sigma_{B}^{2}+\epsilon }}\right)+\beta$$

(6) $$\mathrm{Dropout}\left(x\right)=x\cdot \mathrm{Bernoulli}\left(p\right)$$

here, $\mu_{B}$ and $\sigma_{B}^{2}$ represent the batch mean and variance, respectively; γ and β are learnable parameters; ϵ is a small constant added for numerical stability; and $\mathrm{Bernoulli}\left(p\right)$ is a binary random variable that retains activation with a probability of p.

Table 1 presents the input and output parameters of each layer to provide a clearer understanding of the network structure.

**Figure 4:**
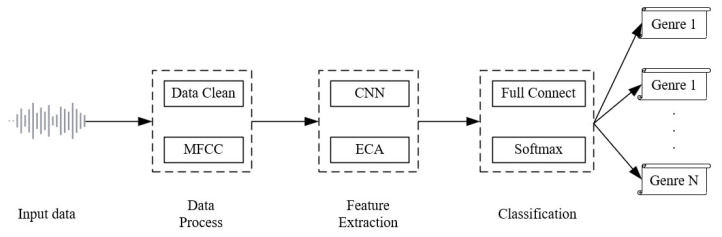
System overall flow chart.

Summarizing the above-described method introduced in this article, Figure 4 lists the overall flow chart of the method used in this article. This flowchart shows each stage of the music genre classification method in detail, covering the complete process from raw audio input to the final classified output. It is divided into three main parts: data processing, feature extraction, and classification.

**Data Processing Stage:**

In this stage, the input audio data are first pre-processed, including data cleaning tasks such as noise removal and signal smoothing. The purpose of this step is to improve the accuracy of subsequent processes and ensure that the extracted features are as precise as possible. The Mel Frequency Cepstral Coefficient (MFCC) is then used to transform the one-dimensional audio signal into a two-dimensional feature map. MFCC is a commonly used technique for audio feature extraction, mapping the frequency features of the audio onto the Mel scale and allowing the neural network to better capture and process the audio signal’s feature information.

**Feature Extraction Stage:**

In this stage, the key features of the input audio are extracted. A convolutional neural network (CNN) is employed to extract the local features of the music signal, such as rhythm, melody, and harmony, layer by layer from the MFCC feature map. The multi-layer convolution operations of the CNN are effective in capturing complex patterns within the audio. Simultaneously, the Efficient Channel Attention (ECA) module is introduced, which adaptively adjusts the weight of each channel to emphasize critical features and suppress irrelevant information. The addition of the ECA module further enhances the effectiveness of feature extraction, enabling the model to more precisely capture key features of the music, particularly when dealing with complex or diverse music genres.

**Classification Stage:**

After feature extraction is completed, the model proceeds to the classification stage. The extracted feature vectors are fed into a fully connected layer, where they undergo further integration and processing via linear transformations. Subsequently, the integrated features are passed into a Softmax classifier, which converts the model’s output into a probability distribution over the music genres. Based on these probabilities, the model ultimately outputs the genre classification result of the audio signal, ranging from genre 1 to genre N.

## 4. Experiments

### 4.1. Experimental Environment Platforms and Datasets

Table 2 describes the environment platform, including both the hardware and software components.

In this study, the GTZAN dataset was employed for the experimental validation of the music genre classification task. The GTZAN dataset is one of the most widely used standard datasets in the field of music information retrieval, originally compiled by Tzanetakis and Cook in 2002 [2]. This dataset contains audio samples from 10 different music genres, with each sample being a 30-s-long audio file, sampled at 22,050 Hz with 16-bit resolution. Each genre in the dataset is represented by 100 samples, ensuring balanced representation across genres, making it ideal for evaluating and comparing music classification algorithms. The audio samples in the GTZAN dataset span the following 10 music genres: blues, classical, country, disco, hip-hop, jazz, metal, pop, reggae, and rock. These samples demonstrate notable differences in rhythm, harmony, and melody, providing rich and diverse features for genre classification tasks. Additionally, as each sample maintains consistent audio duration and sampling parameters, the dataset is highly standardized, facilitating comparative experiments between different algorithms under the same conditions.

### 4.2. Evaluation Indicators

Table 3 presents the definition of the confusion matrix, where Accuracy, Precision, Recall, and F1-Score are used as evaluation metrics for the classification methods discussed in this paper.

Accuracy is the proportion of correctly predicted instances (both positive and negative) out of the total number of instances. It is the most straightforward performance metric.

(7) $$\mathrm{Accuracy}=\frac{\mathrm{TP}+\mathrm{TN}}{\mathrm{TP}+\mathrm{TN}+\mathrm{FP}+\mathrm{FN}}$$

Precision is the ratio of correctly predicted positive instances to all instances predicted as positive. It reflects how many of the instances predicted as positive are actually correct.

(8) $$\mathrm{Precision}=\frac{\mathrm{TP}}{\mathrm{TP}+\mathrm{FP}}$$

Recall: Recall, also known as sensitivity, is the ratio of correctly predicted positive instances to all actual positive instances. It measures the model’s ability to capture positive samples.

(9) $$\mathrm{Recall}=\frac{\mathrm{TP}}{\mathrm{TP}+\mathrm{FN}}$$

The F1-Score is the harmonic mean of Precision and Recall, providing a balanced evaluation of both metrics. The F1-Score is particularly useful when there is a need to balance Precision and Recall.

(10) $$F1-\mathrm{Score}=2\cdot \frac{\mathrm{Precision}\cdot \mathrm{Recall}}{\mathrm{Precision}+\mathrm{Recall}}$$

### 4.3. Results and Analysis

**Figure 5:**
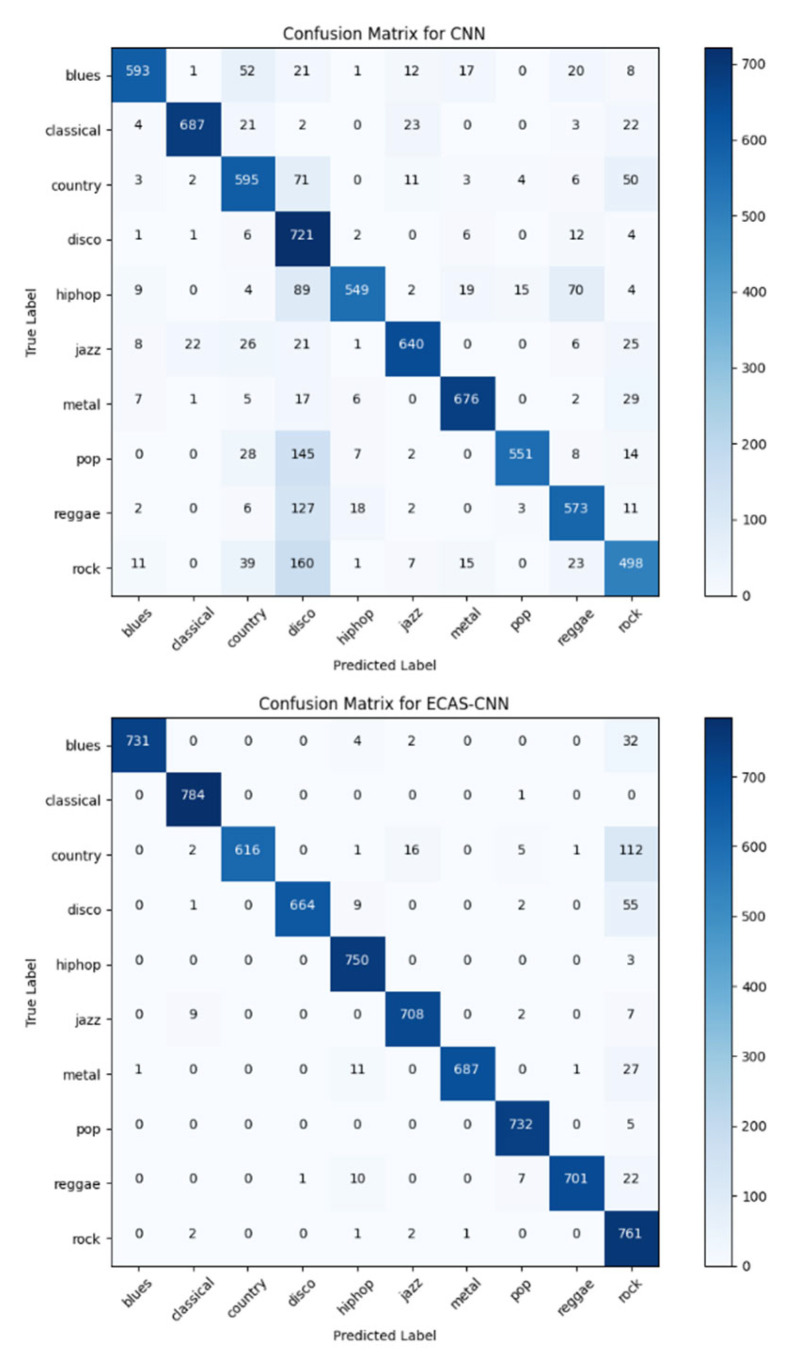
Comparison of Confusion Matrices between CNN and ECAS-CNN.

For the dataset, 80% of the samples from each music genre were randomly selected as the training set, while the remaining 20% were used as the test set. This paper conducts a comparative analysis between the proposed ECAS-CNN algorithm and a standard CNN algorithm, resulting in a comparison of their confusion matrices as well as their PR and Loss curves during training. Figure 5 illustrates the comparison of confusion matrices for the two methods.

The comparison between the confusion matrices clearly demonstrates that the proposed ECAS-CNN model outperforms the traditional CNN model in classifying most music genres. Specifically, in the “blues” category, ECAS-CNN correctly identified 731 samples, a significant improvement over the 593 samples identified by the traditional CNN model. In the “rock” category, ECAS-CNN reduced the number of misclassifications from 160 (as seen in the traditional CNN) to just 22, showcasing its superior accuracy and remarkable ability to minimize misclassifications.

Further analysis reveals that ECAS-CNN exhibits higher accuracy across nearly all music genres. This improvement is particularly evident in genres with complex rhythms and rich harmonies, where capturing intricate musical features is challenging and often leads to classification errors. By leveraging an adaptive channel attention mechanism, ECAS-CNN effectively enhances its ability to capture these critical musical features, thereby reducing inter-class confusion.

Overall, these results indicate that ECAS-CNN not only achieves higher accuracy in music genre recognition tasks but also demonstrates better generalization ability and efficiency. This enhanced classification performance is of significant importance for practical applications in music information retrieval and recommendation systems.

**Figure 6:**
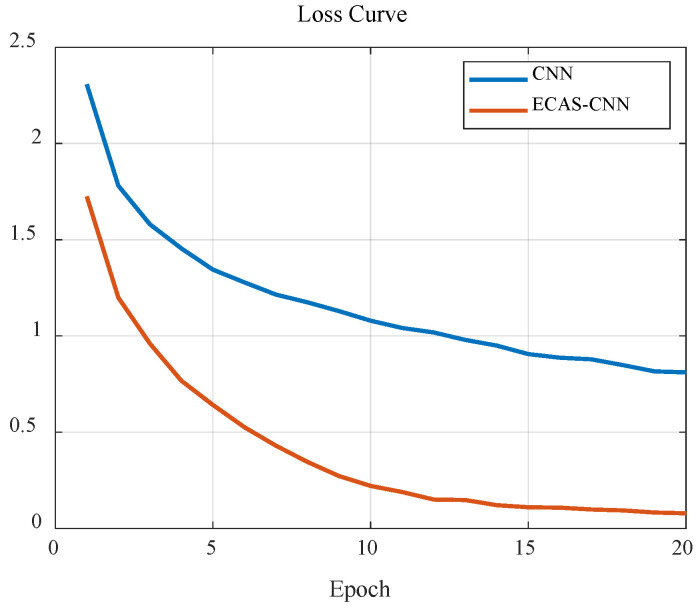
Comparison of Loss Curves.

Figure 6 illustrates the comparison of loss curves between the traditional CNN model and the proposed ECAS-CNN model during the training process. It is evident from the figure that the ECAS-CNN consistently maintains a lower loss value throughout the training process. This lower loss value indicates that ECAS-CNN is more efficient in learning from the data, allowing it to converge to a lower error rate more quickly.

Notably, in the later stages of training, the loss value of ECAS-CNN decreases more significantly, highlighting the advantages of its improved network structure, particularly the introduction of the channel attention mechanism. This mechanism enables the model to more precisely capture complex musical features, contributing to better performance on the training set. Moreover, this significant reduction in loss suggests that the model may possess better generalization capability to new data—a critical attribute for machine learning models to maintain strong performance when faced with unseen data.

Overall, ECAS-CNN demonstrates superior performance compared to the standard CNN model throughout the entire training process, further validating the advantages of its design. Its excellent handling of complex musical signals indicates that ECAS-CNN holds greater potential and application value in music classification tasks. By effectively reducing the loss during training, ECAS-CNN not only improves model accuracy but also enhances training efficiency and final predictive performance.

**Figure 7:**
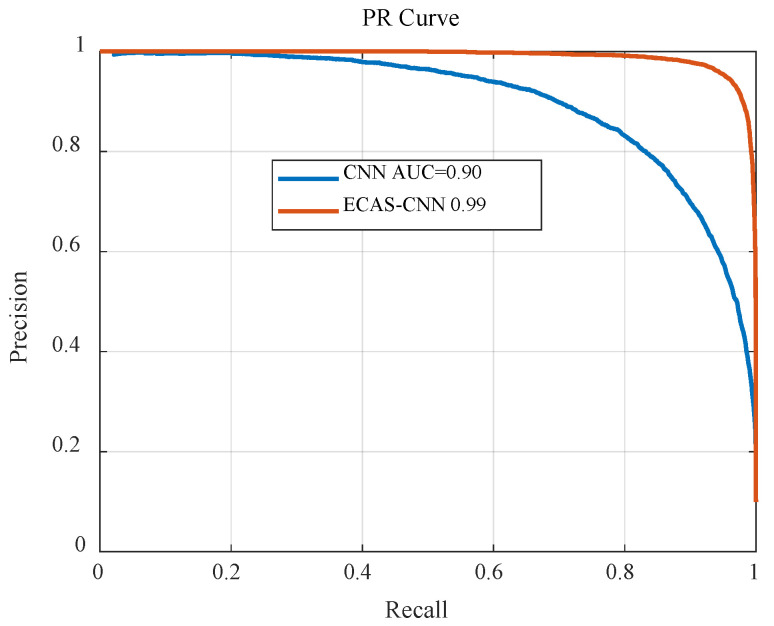
Comparison of Precision-Recall Curves.

Figure 7 presents a comparison of the Precision-Recall (PR) curves between the traditional CNN model and the proposed ECAS-CNN model. It is evident from the figure that the ECAS-CNN significantly outperforms the traditional CNN model across the entire PR curve. Specifically, the PR curve of ECAS-CNN consistently lies above that of the CNN, indicating that for the same recall rate, ECAS-CNN achieves a higher precision. This advantage suggests that ECAS-CNN is more effective in identifying relevant features when processing complex musical signals, leading to more accurate classification.

More importantly, the Area Under the Curve (AUC) for the ECAS-CNN model reaches 0.99, whereas the AUC for the traditional CNN model is only 0.90. This difference further demonstrates the substantial superiority of ECAS-CNN in overall classification performance. The closer the AUC value is to 1, the better the model is at balancing precision and recall across various thresholds, indicating that ECAS-CNN not only excels under specific conditions but also exhibits robust performance across a wide range of potential applications.

Moreover, this improvement in performance is not just theoretical but has significant practical implications. In the context of music classification tasks, a model with higher precision and better recall can greatly enhance the user experience in music recommendation systems while reducing the likelihood of misclassification. This, in turn, increases the overall reliability of the system and boosts user satisfaction.

Additionally, this paper compares the results obtained on the GTZAN dataset with those reported by other studies, as shown in Table 4.

The performance comparison presented in Table 4 clearly indicates that the proposed ECAS-CNN demonstrates significant superiority on the GTZAN dataset. Specifically, ECAS-CNN excels across all four key metrics: Accuracy, Precision, Recall, and F1-Score. The model achieves an accuracy of 95.26%, a precision of 96.22%, a recall of 95.23%, and an F1-Score of 95.41%. These results not only underscore the robust performance of the ECAS-CNN but also highlight its ability to effectively balance different performance metrics, ensuring well-rounded excellence.

In contrast, while the BiLSTM model shows a slightly higher accuracy at 97.80%, its F1-Score is only 78%, reflecting a significant imbalance between precision and recall. This imbalance could lead to suboptimal performance in scenarios where precise classification is critical. Additionally, the 4 Layers-2D CNN model, although relatively balanced in terms of accuracy and F1-Score, falls short in both precision and recall compared to ECAS-CNN, indicating a limitation in its ability to capture complex musical features.

Furthermore, despite the RCNN achieving a high precision of 97.94%, its overall accuracy is the lowest among all compared models, at just 69.49%. This suggests that while RCNN can accurately identify certain samples, its overall classification performance is inadequate, particularly when faced with diverse musical data.

In addition, we also performed MIR classification on the ISMIR dataset, and the results are compared with the most advanced results at present in Table 5.

Experiments on ISMIR 2004 dataset demonstrated that our proposed ECAS-CNN model surpassed other comparative methods across all performance metrics. Specifically, the ECAS-CNN model achieved an accuracy of 94.28%, precision of 95.11%, recall of 94.62%, and an F1 score of 95.23%. These results significantly surpassed those of other methods, such as Deep Learning BAG (92.38% accuracy) and WVG-ELNSC (92.89% accuracy). In comparison, TSVM exhibited relatively weaker performance, with an accuracy of 85.05%. By incorporating the channel attention mechanism (ECA module), ECAS-CNN was able to more effectively extract key features from music signals, resulting in higher accuracy and robustness in complex music genre classification tasks. Overall, ECAS-CNN achieved the best results across all metrics, further confirming the advantages of this approach in music genre classification tasks.

Overall, the notable advantage of ECAS-CNN across multiple performance metrics establishes it as the preferred model for music genre classification tasks. It not only delivers high-precision classification results but also effectively balances all performance indicators, ensuring the model’s stability and reliability in practical applications.

**Complexity Analysis**

Although the ECAS-CNN model exhibits outstanding performance in accuracy and classification capability, its computational complexity warrants further consideration. Compared to traditional CNN models, ECAS-CNN improves feature extraction efficiency by incorporating the ECA module, which adaptively adjusts the attention to each channel in the convolutional layers. However, this adaptive attention mechanism incurs additional computational overhead, particularly when processing large-scale datasets, potentially affecting the model’s training time and resource demands.

Source of Computational Complexity:

The computational complexity of the ECA module primarily stems from the adaptive adjustment of convolution kernel sizes and the weight assignment for each channel. Although the ECA module reduces the number of parameters compared to the SE module (Squeeze-and-Excitation module), it still requires extra channel attention computations after each convolutional layer.

Solutions to Address Complexity:

Model Compression: Techniques such as model pruning, quantization, and distillation can be applied to reduce computational complexity. Through pruning or quantization, the number of model parameters and computational demands can be significantly reduced without notably affecting classification accuracy.

Distributed Computing and Parallelization: In large-scale dataset training, distributed computing or parallel processing can effectively expedite the training process. By leveraging multi-GPU or TPU clusters, training time can be significantly shortened, thereby alleviating the burden of high computational complexity.

Automated Hyperparameter Tuning: Tools such as Bayesian Optimization or Grid Search can be used to further optimize model architecture and training parameters, enhancing performance while reducing complexity.

## 5. Conclusions

In this study, we have proposed the ECAS-CNN model, which demonstrates exceptional performance in the task of music genre classification. The key points of our findings are as follows:

(1) Integration of Channel Attention Mechanism: By integrating an effective channel attention mechanism into the traditional CNN architecture, ECAS-CNN enhances feature extraction capabilities, leading to superior classification performance.
(2) High Performance on the GTZAN Dataset: The ECAS-CNN model achieved a high accuracy of 95.26% on the GTZAN dataset, along with a precision of 96.22% and a recall of 95.23%. These metrics collectively reflect the model’s outstanding classification ability.
(3) Comparison with Advanced Models: When compared to other advanced models such as BiLSTM and 2D CNN, ECAS-CNN exhibited balanced and efficient performance across all key metrics, including accuracy, precision, recall, and F1-Score.
(4) Effectiveness in Handling Complex Features: The results validate the effectiveness of ECAS-CNN in processing complex musical features and improving classification performance, particularly in reducing misclassification and enhancing the model’s generalization capability.

**Future Work**

Model Optimization and Extension: Future work will focus on further optimizing the ECAS-CNN model, particularly in balancing computational complexity and classification performance. We plan to introduce additional lightweight attention mechanisms and more efficient optimization algorithms to enhance the model’s scalability on large-scale datasets.

Integration of Multimodal Data: Future research could combine ECAS-CNN with other data modalities (such as lyrics text and audio emotion data) to build more sophisticated multimodal music classification models. This approach will help improve classification accuracy, particularly in tasks requiring the distinction of musical emotions and styles.

Cross-Dataset Testing and Applications: In the future, we will seek to test the model’s adaptability on other publicly available music datasets to validate its generalization across different music genres and diverse musical styles. Additionally, the model’s application will be extended to real-world music recommendation systems and audio analysis platforms to further validate its practicality and scalability.

## Figures and Tables

**Table 1:** Summary of the ECAS-CNN model.

	Layer	Feature Map	Size	Kernel Size	Stride	Activation
Input	MFCC	1	(130, 13, 1)	-	-	-
1	Conv	128	(130, 13, 128)	(3, 3)	(1, 1)	Relu
2	ECA	128	(130, 13, 128)	-	-	-
3	Max Pool	128	(65, 7, 128)	(2, 2)	(2, 2)	
4	Conv	128	(65, 7, 128)	(3, 3)	(1, 1)	Relu
5	ECA	128	(65, 7, 128)	-	-	-
6	Max Pool	128	(17, 2, 256)	(2, 2)	(2, 2)	-
7	Conv	256	(17, 2, 256)	(3, 3)	(1, 1)	Relu
8	ECA	256	(17, 2, 256)	-	-	-
9	Max Pool	256	(9, 1, 256)	(2, 2)	(2, 2)	-
10	Flatten	-	2304	-	-	-
11	FC	-	128	-	-	Relu
12	Dropout	-	128	-	-	-
13	FC	-	10	-	-	Softmax

**Table 2:** Experimental environment platform.

Character Radical	Model Parameters
CPU	13th Gen Intel(R) Core(TM) i9-13900KF
RAM	32 G
GPU	NVIDIA GeForce RTX 4060 12 GB
Programing Language	Python 3.8
Deeplearning Framework	Tensorflow 2.13.0
CUDA	11.8

**Table 3:** Confusion matrix definition.

		True Value	
		Positive	Negative
Predicted value	Positive	TP	FP
	Negative	FN	TN

**Table 4:** Performance of Different Methods on the GTZAN Dataset.

Method	Accuracy (%)	Precision (%)	Recall (%)	F1-Score (%)
**RCNN with Data Augmentation [18]**	69.49	97.94	88.84	92.7
**4 Layers-2D CNN with Data Augmentation [18]**	81.55	83.50	89.01	86.17
**BiLSTM [19]**	97.80	88	94	78
**MFCC+STFT [20]**	95.20	95.23	95.20	95.20
**CNN-5+DPA [21]**	91.40	90.60	96.00	93.20
**Purposed ECAS-CNN**	95.26	96.22	95.23	95.41

**Table 5:** Performance of Different Methods on the ISMIR 2004 Dataset.

Method	Accuracy (%)	Precision (%)	Recall (%)	F1-Score (%)
**Deep Learning BAG**	92.38	91.83	92.55	92.03
**TSVM**	85.05	84.68	85.12	84.80
**WVG-ELNSC**	92.89	91.55	92.62	89.87
**RA based TSM-SVM [22]**	91.84	90.56	91.23	89.51
**Purposed ECAS-CNN**	94.28	95.11	94.62	95.23

## Data Availability

Data are contained within the article.
